# Effect of Medication Intake on Dental Implant‐Related Outcomes—A Scoping Review

**DOI:** 10.1111/clr.70151

**Published:** 2026-07-05

**Authors:** Alberto Monje, Maria Costanza Soldini, Emilio Couso‐Queiruga, Álvaro Babiano, Vivianne Chappuis

**Affiliations:** ^1^ Department of Periodontology Universitat Internacional de Catalunya Barcelona Spain; ^2^ Department of Periodontology University of Bern Bern Switzerland; ^3^ Department of Oral Surgery and Stomatology University of Bern Bern Switzerland

**Keywords:** dental implant, endosseous implant complication, mucositis, peri‐implant disease, peri‐implantitis

## Abstract

**Objective:**

The objective of this scoping review was to evaluate the effect of systemic medications on dental implant outcomes in medically compromised patients.

**Material and Methods:**

Electronic and manual searches were conducted for this scoping review. Clinical studies reporting on systemic medication intake and implant outcomes were eligible. Studies reporting on the use of antiresorptive medications were not included in this scoping review. Qualitative evaluation was performed as meta‐analysis was not feasible due to study heterogeneity.

**Results:**

Overall, 51 studies met the inclusion criteria and were eligible for assessment. Six medications were identified to potentially impact the short‐ and long‐term implant outcomes. In total, 8 studies reported the intake of anti‐hypertensives (AHTNs), 9 studies reported the intake of anticoagulants (ACs), 12 studies reported the intake of immunosuppressants (IMNs), 11 studies reported the intake of nonsteroidal anti‐inflammatories (NSAIDs), 12 studies reported the intake of proton‐pump inhibitors (PPIs), and 12 studies reported the intake of selective serotonin reuptake inhibitors (SSRIs). Moreover, 1 study reported pooled data regarding the use of PPIs, SSRIs, AHTNs, and statins. From these, the following results were drawn: ACs, long‐term NSAID use, prolonged use of PPIs, and SSRIs are associated with increased risk of implant failure and/or marginal bone loss. Additionally, AHTNs and IMNs have no effect on implant‐related outcomes. Medication‐related biological implant complications are underreported in the literature.

**Conclusion:**

Medication intake can significantly influence implant‐related outcomes.

## 1 Introduction

The widespread application of implant therapy as an alternative to restore function and esthetics in edentulous ridges has led to the use of dental implants in systemically compromised patients or under chronic intake of medication for the treatment of disabling diseases. These scenarios became more common in the daily clinical practice due to the increase in life expectancy in developed/emerging countries and in the overall increase in global population of 50 million annually (Srinivasan et al. 2017). The deleterious impact of uncontrolled medical conditions, such as diabetes mellitus, hyperlipidemia, rheumatoid arthritis, or osteoporosis, amongst many others, has been described in the literature (Insua et al. 2024). The link between chronic conditions and implant failure relies on the induction of a low‐grade systemic inflammatory condition associated with high levels of circulating pro‐inflammatory cytokines that favor the chemotaxis and activation of monocytes, neutrophils, and adipose tissue macrophages (Hill et al. 2014). These may ultimately contribute to an altered homeostatic process that may lead to early implant failure or to bone loss subsequently following restoration, increasing the risk of peri‐implant diseases.

Moreover, the systemic intake of medication to manage chronic disorders may further alter bone metabolism. Medications such as nonsteroidal anti‐inflammatory drugs (NSAIDs), selective serotonin reuptake inhibitors (SSRIs), proton pump inhibitors (PPIs), antiresorptive medications, and anti‐hypertensives (AHTNs) were suggested as contributors to modulating bone conditions and the biological response of the alveolar bone to dental implants. Chappuis et al. performed a systematic review commissioned by International Team for Implantology (ITI) and identified 17 studies that demonstrated associations of medications with implant failure (Chappuis et al. 2018). Overall, PPIs and SSRIs used for the treatment of gastroesophageal reflux/gastric ulcers and for depression/anxiety, respectively, were significantly linked to implant failure. These findings were not surprising since PPIs impair the effective calcium uptake through the intestines, and this may negatively impact the bone mineral density. Similarly, SSRIs inhibit the serotonin that regulates osteoclast activation and differentiation, as osteoclasts derive from hematopoietic cell precursors. None of the other medications explored demonstrated significance with implant failure. However, while not notably affecting implant survival, the use of NSAIDs was noticed to be more associated with bone loss (Chappuis et al. 2018). On the other hand, it seems that the use of AHTNs may be beneficial since they inhibit osteoclasts' catabolic effects on bone by blocking the β2 adrenergic receptors or by shifting the balance toward bone formation by blocking the renin‐angiotensin system (Insua et al. 2024).

Given the light that these findings shed, the interest in research within this subject has experienced notable growth. Therefore, the objective of this scoping review was to provide an update on the impact of medications used on implant outcomes, including implant failure and biological complications, in medically compromised patients.

## 2 Material and Methods

This review was conducted following the guidelines of the Preferred Reporting Items for Systematic Reviews and Meta‐Analyses extension for Scoping Reviews (PRISMA‐ScR) (Tricco et al. 2018). Due to the substantial heterogeneity and the limited quantity of available evidence on this subject, conventional data synthesis methods typically used in systematic reviews and meta‐analyses were not feasible. As a result, a scoping approach was adopted to comprehensively map the existing literature and evaluate the effects of concomitant medications (AHTNs, anticoagulants (ACs), immunosuppressants (IMNs), NSAIDs, PPIs, and SSRIs) on dental implant failure and biological complications.

### 2.1 Research Question

In patients receiving dental implants, how does the use of ACs and/or AHTNs and/or IMNs and/or NSAIDs and/or PPIs and/or SSRIs affect implant survival and biological outcomes compared to patients not taking these medications?

### 2.2 PECO Question

– Population: Edentulous or partially edentulous patients rehabilitated with dental implants
– Exposure: Use of medications (e.g., AHTNs, ACs, IMNs, NSAIDs, PPIs, and SSRIs) for chronic disorders.
– Comparison: Patients not taking medications in the presence or absence of chronic disorders or taking other medications.
– Outcome: Implant failure and biological complications.

### 2.3 Search Strategy

An electronic literature search was performed in MEDLINE (PubMed) and EMBASE up to July 21st, 2025, by two examiners (MCS and AB). The search strategy incorporated both MeSH terms and free‐text keywords. Filters were applied to include only studies published in English and restricted to human subjects. The complete search strategy used for MEDLINE (PubMed) is provided in Appendix S1. A manual search was further performed from the bibliography of included and excluded articles. In addition, a manual search was performed by reviewing the reference lists of the included studies.

### 2.4 Eligibility Criteria

- Human clinical studies (prospective or retrospective cohort studies, randomized controlled trials (RCT), observational studies, or case series).
- Studies investigating the effect of at least one concomitant systemic medication, including: AHTNs, ACs, IMNs, NSAIDs, PPIs and SSRIs on implant therapy. Antiresorptive medications were excluded as this topic was addressed in a recent publication (Mertens et al. 2025).
- Studies reporting at least one of the following outcomes: implant failure, marginal bone loss or biological complications (e.g., peri‐implant mucositis).

### 2.5 Study Selection

After eliminating duplicate entries, an initial title screening was conducted by a single reviewer (MCS) to identify potentially relevant studies. Abstracts and full texts were subsequently reviewed independently by two calibrated reviewers (MCS and AB). For articles lacking abstracts, full‐text evaluation was performed directly. Any disagreements encountered during the screening process were discussed until consensus was reached. The reasons for exclusion at the full‐text stage were systematically documented.

### 2.6 Data Extraction

Using a standardized data extraction sheet, one reviewer (MCS) systematically collected the following information from each included full‐text article: authorship, year of publication, study design, patient sample size, gender distribution, number of dental implants, systemic condition, type of medication, dosage (mg/ml), duration of therapy (months), implant failure rate and timing (early or late), occurrence of peri‐implant mucositis, peri‐implantitis, peri‐implant bone loss, and any reported adverse events.

### 2.7 Methodological Quality and Risk of Bias Assessment

The methodological quality of the included studies was assessed using tools appropriate to their study design. For randomized controlled trials, we applied the Risk of Bias 2 tool (RoB 2, 2019 version), which is specifically designed to evaluate potential bias across domains such as the randomization process, deviations from intended interventions, missing outcome data, outcome measurement, and selection of the reported results.(J. A. C. Sterne et al. 2019) For nonrandomized studies, we used the ROBINS‐I tool, version 2, which provides a structured framework for assessing bias in studies that do not employ random allocation, including potential confounding, selection bias, classification of interventions, deviations from intended interventions, missing data, measurement of outcomes, and selection of the reported results.(J. A. Sterne et al. 2016).

## 3 Results

### 3.1 Study Selection

**FIGURE 1:**
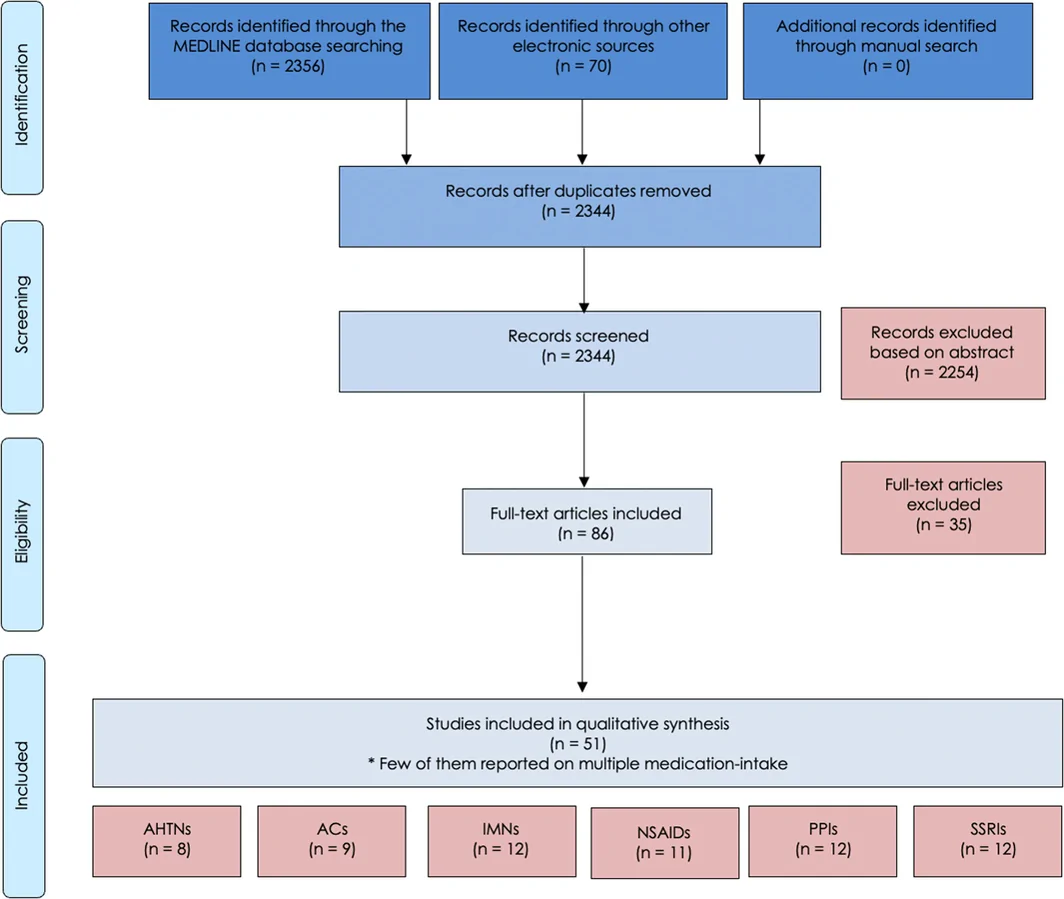
PRISMA flowchart.

**FIGURE 2:**
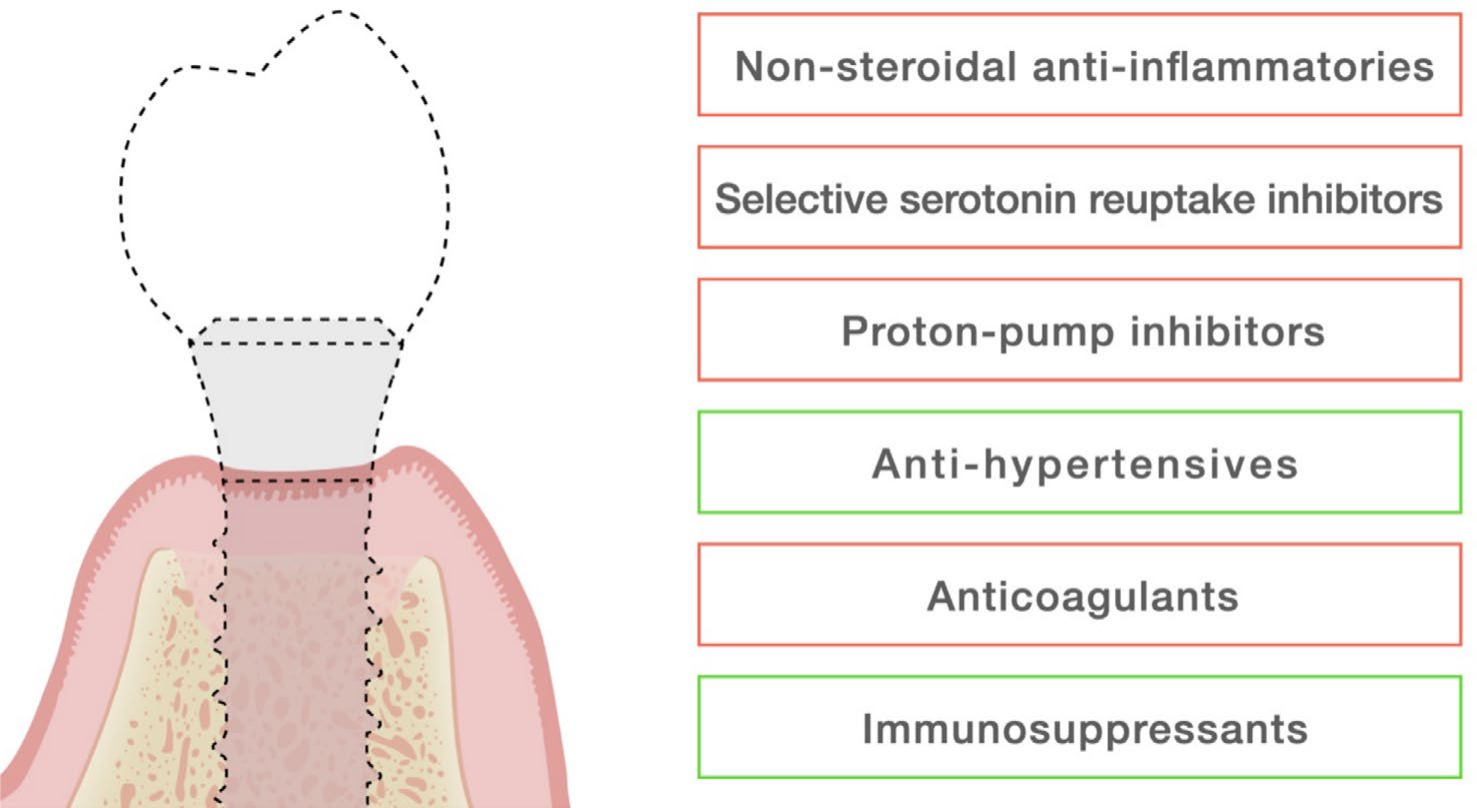
Effects of medication on implant failures. Medications with a deleterious effect are represented by red squares, whereas those with a beneficial effect are represented by green squares.

The initial search identified 2356 potentially relevant records (Figure 1). After removing duplicates, 2344 articles remained for screening. Following the title and abstract evaluation, 86 articles were selected for full‐text review, resulting in 51 studies being included in the final assessment. Of these, 2 articles were case series studies (Gandhi and Bhatavdekar 2019; Gu and Yu 2011), 33 articles were retrospective studies (8–40, 42), 4 articles were case–control studies (Basson et al. 2023; D'Orto et al. 2024; El‐Sherbini 2018; M. Tattan et al. 2021), 1 article was a cross‐sectional study (Romandini et al. 2021), 5 articles were prospective studies (Hernandez et al. 2019; Khamis et al. 2019; Montebugnoli et al. 2015; Paredes et al. 2018; Radzewski and Osmola 2019), and 6 articles were RCT studies (41, 52–56). The included studies were grouped according to medication categories and are summarized in Table 1. Table 2 includes the biological plausibility of medication‐related implant outcomes, and Figure 2 includes the effect of medication on implant failures. Due to the heterogeneity of the original studies, meta‐analysis was not deemed feasible.

**TABLE 1:** Included clinical studies reporting on medication intake and implant outcomes.

Author (year)	Study design	Patients (N)	Implants (N)	Systemic condition	Medication	Dosage (mg/mL)	Therapy length (months)	Failure rate patient level (%)	Failure rate implant level (%)	Implant failure timing (early/late)	Peri‐implant mucositis (%)	Peri‐implantitis (%)	MBL (mm) (SD)	Adverse events	Concluding remarks
Alissa et al. (2009)	RCT	29	41	ASA I–II	NSAIDs (Ibp)	600 mg	4× day 7 days	0	0	Early	NA	NA	1.1 ± 1	No	Short course of systemic ibuprofen for postoperative pain control following dental implant placement may have no significant impact on marginal bone levels around implants during the early healing phase
		29	48		—	—	—	0	0	Early	NA	NA	1.2 ± 1		
Anitua et al. (2018)	RC	23	66	LP	IMNs	7.5–30 mg	8 days	4.3	1.5	Late	NA	NA	Mesial: 1 ± 0.9 Distal: 1 ± 1.1	NA	Stable long‐term outcomes can be expected for short implants placed in patients with oral lichen planus and taking glucocorticoids to control the disease.
Altay et al. (2018)	RC	36	109	DC	SSRIs	NA	NA	5.6	1.8	Early	NA	NA	NA	NA	Intake of SSRIs leads to increase in the rate of osteointegration failure, although not reaching a statistically significant level. Odds of osseointegration failure were 3.12 times greater for SSRI users
		595	1946	ASA I–II	—	—	—	1.8	0.6	Early	NA	NA	NA		
Altay et al. (2019)	RC	24	69	GD	PPIs	NA	NA	8.3	NA	Early	NA	NA	NA	NA	Dental implants placed in patients using PPIs were found to be 4.30 times more likely to fail prior to loading
		568	1849	ASA I–II	—	—	—	1.9	NA	Early	NA	NA	NA		
Basson et al. (2023)	CC	1171	NA	ASA I–II, DB, OST, HTA, IBD	NSAIDs	NA	≥ 6	NA	NA	Early	NA	NA	NA	NA	PPI, ATHA, immunosuppressive medications (corticosteroids), NSAIDs, and SSRIs were not associated with early implant failure
					—	—	—	NA	NA	Early	NA	NA	NA		
					IMNs	NA	≥ 6	NA	NA	Early	NA	NA	NA		
					—	—	—	NA	NA	Early	NA	NA	NA		
					SSRIs	NA	≥ 6	NA	NA	Early	NA	NA	NA		
					—	—	—	NA	NA	Early	NA	NA	NA		
					ATHAs	NA	≥ 6	NA	NA	Early	NA	NA	NA		
					—	—	—	NA	NA	Early	NA	NA	NA		
					PPIs	NA	≥ 6	NA	NA	Early	NA	NA	NA		
					—	—	—	NA	NA	Early	NA	NA	NA		
Block and Mercante (2025)	RC	97	232	ASA I, II, III	SSRIs	NA	NA	18.6	22	Early	NA	NA	NA	NA	Patients who use SSRI at the time of implant surgery may have an increased risk for implant failure. Patients taking SSRI present a RR = 2.8 (95% CI: 1.8–4.4)
		1514	2496	NA	—	—	—	6.7	9	Early	NA	NA	NA		
Brizuela‐Velasco et al. (2021)	RC	110	46	NA	ACs	NA	NA	NA	4.5	Early	NA	NA	NA	NA	The risk of loss was multiplied by 28.2 for patients treated with anticoagulants. Patients treated with AC suffered from some other systemic pathology and were older than the mean age. Age and comorbidities seem to be stronger determining factors for implant success and survival than the administration of specific drugs.
								NA	11.4	Late	NA	NA	NA		
			251	NA	—	—	—	NA	2.0	Early	NA	NA	NA		
								NA	1.2	Late	NA	NA	NA		
Carr et al. (2019)	RC	603	NA	NA	SSRIs	NA	NA	10.4	NA	Early/late	NA	NA	NA	NA	Active use of SSRIs did not significantly increase the risk of implant failure
		4853	NA	NA	—	—	—	9.6	NA	Early/late	NA	NA	NA	NA	
Chandra et al. (2021)	RC	128	245	DC	SSRIs	NA	NA	NA	12	Early/late	NA	NA	NA	NA	SSRIs is associated with an increased rate of implant failure.
		282	475	ASA I–II	—	—	—	NA	5.8	Early/late	NA	NA	NA		
Chaushu et al. (2023)	RC	173	708	IHD, AF/VF, CVA	ACs	NA	NA	18.5	5.5	Early	NA	NA	NA	NA	At the implant level, the use of AC was found to be associated with increased odds of early implant failure (OR = 2.64). At the patient level, no significant relationship was found between anticoagulant use and implant failure when controlling for other variable.
		514	2263	ASA I–II–III	—	—	—	14	3.3	Early	NA	NA	NA		
Chatzopoulos and Wolff (2025a)	RC	1332	2872	NA	PPIs	NA	NA	8.6	6.8	Early/late	NA	NA	NA	NA	Patients using PPIs exhibited a significantly increased risk of dental implant failure. Omeprazole users showed a significantly increased risk for implant failure (HR = 1.45) compared to nonusers.
Chatzopoulos and Wolff (2025b)	RC	1985	4770	NA	Antiplatelet	NA	NA	2.9	1.6	Early/late	NA	NA	NA	NA	The implant treatment outcome was not significantly associated with the use of any of these two types of medications
		380	NA	NA	ACs	NA	NA	3.2	1.3	Early/late	NA	NA	NA		
		18,857	45,563	NA	—	—	—	NA	1.4	Early/late	NA	NA	NA		
Chrcanovic et al. (2017a)	RC	18	48	DC	SSRIs	NA	NA	NA	12.5	Early/late	NA	NA	NA	NA	SSRIs may not be associated with an increased risk of dental implant failure.
		282	883	ASA I–II	—	—	—	NA	3.3	Early/late	NA	NA	NA		
Chrcanovic et al. (2017b)	RC	67	250	NA	PPIs	NA	≥ 12 weeks	NA	2.4	Early	NA	NA	NA	NA	The intake of PPIs was shown to have a statistically significant negative effect for implant survival rate (HR 2.81; [95% CI: 1.139, 6.937])
								NA	9.6	Late					
		932	3309	NA	—	—	—	NA	1.2	Early	NA	NA	NA		
								NA	3.3	Late					
Chrcanovic et al. (2016)	RC	994	249	NA	PPIs	NA	NA	NA	12	Early/late	NA	NA	NA	NA	Significant difference of the implant survival between implants placed in PPIs users and nonusers (OR: 2.9 [95% CI: 1.9, 4.3])
			3300	NA	—	—	—	NA	4.5	Early/late	NA	NA	NA		
			1150	NA	AHTNs	NA	NA	NA	4.4	Early/late	NA	NA	NA		
			2399	NA	—	—	—	NA	5.3	Early/late	NA	NA	NA		
			52	NA	IMNs	NA	NA	NA	13.5	Early/late	NA	NA	NA		
			3497	NA	—	—	—	NA	4.9	Early/late	NA	NA	NA		
			342	NA	Antidepressant	NA	NA	NA	9.4	Early/late	NA	NA	NA		
			207	NA	—	—	—	NA	4.6	Early/late	NA	NA	NA		
Chrcanovic et al. (2017c)	RC	**The intake of medicaments to reduce the acid gastric production is a negative factor exerting a significant influence on patients having at least three dental implant failures (OR: 1.890 [95% CI: 1.105, 3.233])**													
		142	NA	NA	Antidepressant	NA	NA	54.2	NA	Early/late	NA	NA	NA	NA	
		330	NA	NA	—	—	—	54.5	NA	Early/late	NA	NA	NA		
		69	NA	NA	PPIs	NA	NA	52.1	NA	Early/late	NA	NA	NA		
		352	NA	NA	—	—	—	55.4	NA	Early/late	NA	NA	NA		
		17	NA	NA	IMNs	NA	NA	41.2	NA	Early/late	NA	NA	NA		
		415	NA	NA	—	—	—	54.7	NA	Early/late	NA	NA	NA		
		**Patients with 0–2 failures**													
		447	NA	NA	Antidepressant	NA	NA	3.4	NA	Early/late	NA	NA	NA	NA	
		4879	NA	NA	—	—	—	3.0	NA	Early/late	NA	NA	NA		
		412	NA	NA	PPIs	NA	NA	4.1	NA	Early/late	NA	NA	NA		
		4848	NA	NA	—	—	—	2.9	NA	Early/late	NA	NA	NA		
		60	NA	NA	IMNs	NA	NA	1.7	NA	Early/late	NA	NA	NA		
		5230	NA	NA	—	—	—	3.0	NA	Early/late	NA	NA	NA		
Corbella et al. (2022)	RC	26	NA	NA	PPIs	NA	≥ 5 years	NA	NA	NA	NA	NA	NA	NA	The use of anti‐inflammatory medicines produced a significant effect on peri‐implantitis as compared to subjects not using the drug, with a 2.7‐year drop in the mean survival time. In subjects with more than 1‐ year follow‐up no effect of drug use was found on implant survival.
		244	NA	NA	—	—	—	NA	NA	NA	NA	NA	NA		
		24	NA	NA	SSRIs	NA	≥ 5years	NA	NA	NA	NA	NA	NA		
		246	NA	NA	—	—	—	NA	NA	NA	NA	NA	NA		
		69	NA	NA	AHTNs	NA	≥ 5 years	NA	NA	NA	NA	NA	NA		
		201	NA	NA	—	—	—	NA	NA	NA	NA	NA	NA		
		31	NA	NA	Anti‐inflammatory (Asc, Cortisone, other)	NA	≥ 5 years	NA	NA	NA	NA	NA	NA		
		239	NA	NA	—	—	—	NA	NA	NA	NA	NA	NA		
Deepa et al. (2018)	RC	110	230	DC	SSRIs	NA	NA	10.8	5,3	Early/late	NA	NA	NA	NA	Failure rates of dental implants are significantly increased in patients taking SSRIs, especially in patients above 50 years of age
		242	450	NA	—	—	—	4.7	1.5	Early/late	NA	NA	NA		
D'Orto et al. (2024)	CC	28	142	CDV	ACs, AHTNs or AC + AHTNs	NA	NA	NA	1.6	Early	NA	NA	1.0 ± 1.0	Edema: 2 Pain: 3 Bleeding: 8 Wound Infection: 1	No significant differences were observed in the values of implant survival rate, marginal bone loss, peri‐implant parameters, and the incidence of surgical complications concerning drug intake.
								NA	0.8	Late	NA	NA	0.6 ± 0.7		
		26	128	ASA I–II	—	—	—	NA	2.1	Early	NA	NA	1.0 ± 1.0	Edema: 2 Pain: 3 Bleeding: 3 Wound Infection: 1	
								NA	2.1	Late	NA	NA	0.6 ± 0.8		
El‐Sherbini (2018)	CC	7	14	RA	IMNs	10 mg	NA	0	0	Early	NA	NA	NA	NA	Patients with rheumatoid arthritis taking prednisolone present high implant success rate
		7	14	NA	—	—	—	0	0	Early	NA	NA	NA		
Galletti et al. (2020)	RC	12	57	Nonvalvular AF	ACs	20 mg/day	≥ 6	0.0	0.0	Early/late	NA	NA	NA	– No major postoperative bleeding events were reported. – 25% presented slight immediate postoperative bleeding. – 33.3% suffered from swelling in the first week after surgical procedures	100% survival of multiple implant placement with an immediate loading in patients taking Rivaroxaban
Gandhi and Bhatavdekar (2019)	CS	6	12	NA	ACs	Rivaroxaban 20 mg once a day Apixaban 2.5 mg once twice a day Dabigatran 75 mg once twice a day	Rivaroxaban 12 months Apixaban 18 months Dabigatran 12 months	0.0	0.0	Early	NA	NA	NA	No	Flapless dental implant placement in patients on uninterrupted direct anticoagulant therapy demonstrated favorable primary stability and ISQ values.
Gu and Yu (2011)	CS	13	45	OT	IMNs	NA	NA	0	0	Early/late	NA	NA	1.3 ± 1.2	NA	Dental implant treatment can be placed in patients with liver transplant under in a stable condition with long‐term immunosuppression.
Hakam et al. (2021)	RC	41	NA	ASA I–II	SSRIs	NA	NA	7.3	6.3	Early/late	NA	NA	NA	NA	Users of antidepressants were at higher risk of implant failure than nonusers. Implant failure was associated with the use of SNRI (OR: 11.07) and TCA (OR: 12.17). OR of implant failure for users of SSRI antidepressant did not differ from that of nonusers.
		13	NA		SNRI	NA	NA	30.8	31.3	Early/late	NA	NA	NA		
		4	NA		TCA	NA	NA	25	33.3	Early/late	NA	NA	NA		
		1	NA		MAOI	NA	NA	0	0	Early/late	NA	NA	NA		
		12	NA		Atypical	NA	NA	8.3	5.2	Early/late	NA	NA	NA		
		1	NA		SNRI + TCA	NA	NA	100	100	Early/late	NA	NA	NA		
		5	NA		SSRIs + Atypical	NA	NA	0	0	Early/late	NA	NA	NA		
		2	NA		SNRI + Atypical	NA	NA	0	0	Early/late	NA	NA	NA		
		2	NA		SSRIs + TCA	NA	NA	0	0	Early/late	NA	NA	NA		
		691	NA		—	—	—	3.9	3.9	Early/late	NA	NA	NA		
Hernandez et al. (2019)	PC	25	79	LT	IMNs	NA	NA	0	0	Early/late	46.8	5.1	1.6 ± 1.0	NA	Dental implants in immunosuppressed are shown long‐term follow‐up.
		28	86	ASA I–II	—	—	—	NA	1.1	Early/late	48.8	8.1	1.9 ± 0.8		
Jeffcoat et al. (1995)	RCT	29	29	ASA I–II	NSAIDs (Flb)	50 mg	3 months	0	0	Early	NA	NA	0.6	NA	NSAID flurbiprofen reduce bone loss around implants in the first year of use
					NSAIDs (Flb)	100 mg	3 months	0	0	Early	NA	NA	1.1		
					—	—	—	0	0	Early	NA	NA	1.6		
Jonas et al. (2024)	RC	41	117	IHD, AF/VF, CVA	Antiplatelet	NA	NA	45.5	4.3	Early	NA	NA	NA	NA	AC and Antiplatelet medications are not significant risk factors for EIF following sinus floor augmentation
		31	86	IHD, AF/VF, CVA	ACs	NA	NA	36.4	5.8	Early	NA	NA	NA		
		38	102	ASA 1	—	—	—	18.2	3.9	Early	NA	NA	NA		
Khamis et al. (2019)	PC	20	20	LP	IMNs	4 mg every 48 h	48 months	0	0	Early/late	NA	NA	0.6 ± 0.1	NA	Patients medicated with corticosteroids demonstrated MBL comparable to healthy individuals.
		22	22		IMNs	4 mg every 48 h	12 weeks	0	0	Early/late	NA	NA	2.5 ± 0.2		
		17	17	—	—	—	—	0	0	Early/late	NA	NA	0.6 ± 0.1		
Kotsailidi et al. (2023)	RC	48	76	ASA I–II	SSRIs	NA	NA	0	0	Late	NA	NA	0.4 ± 0.8	NA	SSRIs is associated with greater marginal bone loss around osseointegrated dental implants in function for a mean period of 3.8 year.
		57	76		—	—	—	0	0	Late	NA	NA	0.0 ± 0.6		
Kumchai et al. (2025)	RCT	7	7	ASA I–II	NSAIDs (Npx)	220 mg	3×days 7 days	14.3	14.3	Early	NA	NA	NA	NA	No significant differences in ISQ value or marginal bone loss after up to 16 weeks of follow‐up between subjects from naproxen and placebo groups
		5	5		—	—	—	0	0	Early	NA	NA	NA		
Markovic et al. (2017)	RCT	20	80	NA	ACs	NA	NA	0.0	0	Early	NA	NA	NA	NA	No early or late implant loss was observed among AC users. AC appears to influence the bone healing events resulting in lower ISQ in the end of 3‐month period without compromising implant stability.
								0.0	0	Late	NA	NA	NA		
Masri et al. (2023)	RC	119	555	ASA I–II–III	PPIs	NA	≥ 1year	19.3	5.4	Early	NA	NA	NA	NA	Multivariate analysis yielded statistically significant OR of 1.91 for EIF following PPIs use
		568	2416		—	—	—	14.3	3.5	Early	NA	NA	NA		
Masri et al. (2025)	RC	185	784	HTA	AHTNs	NA	NA	9.7	2.3	Early	NA	NA	NA	NA	Hypertension negatively affects bone formation. Antihypertensive drugs have a positive effect on bone formation and subsequently on early failure.
		14	64	HTA	—	—	—	28.6	6.3	Early	NA	NA	NA		
		593	2123	ASA I–II–III	—	—	—	14.5	4.3	Early	NA	NA	NA		
Montebugnoli et al. (2015)	PC	13	29	OT	IMNs	NA	NA	0	0	Early	NA	NA	0.2 ± 0.1	NA	The bone and periodontal response, as well as the microbiological status around submerged dental implants, appear comparable between immunocompromised patients and healthy controls.
		13	28	ASA I–II	—	—	—	0	0	Early	NA	NA	0.2 ± 0.1		
Paredes et al. (2018)	PC	14	48	LT	IMNs	NA	NA	0	0	Early/late	35.4	4.2	NA	NA	Pharmacologically immunosuppression in liver transplant patients was not a risk factor for implant failure, nor for the incidence of peri‐implant diseases
		16	54	ASA I–II	—	—	—	NA	1.85	Early/late	43.4	9.4	NA		
Petsinis et al. (2017)	RC	2	7	SS	IMNs	5–10 mg	NA	0	0	Early/late	NA	0	NA	NA	Glucocorticosteroid intake for systemic diseases does not have a significant impact on the osseointegration.
		11	37	RA	IMNs	5–60 mg	NA	1.1	2.7	Early/late	NA	2.7	NA		
		3	14	LE	IMNs	10–20 mg	NA	0	0	Early/late	NA	0	NA		
		2	5	GCA	IMNs	10–60 mg	NA	0	0	Early/late	NA	0	NA		
		1	2	PN	IMNs	10–20 mg	NA	0	0	Early/late	NA	0	NA		
		1	3	DM	IMNs	5–10 mg	NA	0	0	Early/late	NA	0	NA		
		2	4	MG	IMNs	10–40 mg	NA	0	0	Early/late	NA	0	NA		
		1	7	PSO	IMNs	10–20 mg	NA	0	14.3	Early/late	NA	0	NA		
Radzewski and Osmola (2019)	PC	21	24	OT	IMNs	NA	24 months	0	0	—	NA	NA	0.3 ± 0.2	NA	Immunosuppressive medications administered to organ transplants patients do not have any impact on the osseointegration of dental implants.
		15	15	ASA I–II	—	—	—	NA	6.7	Early	NA	NA	0.5 ± 0.4		
Reddy et al. (1990)	RCT	2	NA	ASA I–II	NSAIDs (Flb)	100 mg	2× days 4 months	0	0	—	NA	NA	NA	NA	Intake of NSAIDS is associated with increased bone density during the initial phase of healing around dental implants
		2	NA	ASA I–II	—	—	—	0	0	—	NA	NA	NA		
Rodriguez‐Pena et al. (2022)	RC	31	71	ASA I–II	SSRIs	NA	≥ 1 year	NA	18.3	Early/late	NA	NA	NA	NA	SSRIs are associated with a 4.53 times higher rate of dental implants failure
		237	502		—	—	—	NA	4.4	Early/late	NA	NA	NA		
Rogoszinski et al. (2022)	RC	227	323	NA	PPIs	NA	≥ 5 years	NA	18.0	Late	NA	19.1	NA	NA	No associations between PPI use and implant failure or peri‐implantitis.
		393	610	NA	—	—	—	NA	24.0	Late	NA	25.1	NA		
Romandini et al. (2021)	CSECT	4	NA	NA	PPIs	NA	NA	NA	NA	NA	25.0	NA	NA	NA	Multivariate analysis showed that PPI (OR = 0.08 9 [95% CI: 0.01–0.90]), and AC (OR = 0.08 [95% CI: 0.01–0.56]) as protective indicators for peri‐implantitis.
		94	NA	NA	—	—	—	NA	NA	NA	58.5	NA	NA		
		5	NA	NA	ACs	NA	NA	NA	NA	NA	40.0	NA	NA		
		93	NA	NA	—	—	—	NA	NA	NA	58.1	NA	NA		
		5	NA	NA	NSAIDs	NA	NA	NA	NA	NA	60.0	NA	NA		
		93	NA	NA	—	—	—	NA	NA	NA	57.0	NA	NA		
		13	NA	NA	Antidepressant	NA	NA	NA	NA	NA	53.8	NA	NA		
		85	NA	NA	—	—	—	NA	NA	NA	57.6	NA	NA		
Sakka and Hanouneh (2013)	RCT	14	31	ASA I–II	NSAIDs (Ibp)	600 mg	4× days 7days	0	0	Early	NA	NA	0.2 ± 0.3	NA	Short‐term postoperative use of ibuprofen does not significantly affect marginal bone loss or implant osseointegration in healthy patients.
		14	26		—	—	—	0	0	Early	NA	NA	0.3 ± 0.2		
Seki et al. (2020)	RC	13	34	HTA	AHTNs	NA	NA	0	0	Late	20.9	4.7	NA	NA	No implant loss was reported. History of antihypertensive medication use was associated with an OR of 12.90 for peri‐implantitis.
		22	43	—	—	—	—	0	0	Late	17.6	26.5	NA		
Tattan et al. (2021)	CC	201	201	NA	NSAIDs	NA	Long term use	37.1	37.1	Early	NA	NA	NA	NA	Post‐operative NSAID use was associated with early implant failure in the bivariate analysis (HR: 2.8, 95% CI: 1.0–7.7) but loss significance in the multivariate analysis.
					—	—	—	27.6	27.6		NA	NA	NA		
					NSAIDs	NA	Post‐surgery	32.9	32.9		NA	NA	NA		
					—	—	—	12.9	12.9		NA	NA	NA		
					PPIs, SSRIs, AHTNs, statins	NA	NA	30.6	30.6		NA	NA	NA		
					—	NA	NA	30.7	30.7		NA	NA	NA		
Tonini et al. (2022)	RC	120	1887	HTA	AHTNs	NA	NA	7.5	NA	Late	NA	NA	NA	NA	Antihypertensives did not seem to influence the success rate of the implants.
		51		HTA	—	—	—	5.9	NA	Late	NA	NA	NA		
		423		HTA	—	—	—	7.0	NA	Late	NA	NA	NA		
Urdaneta et al. (2011)	RC	13	61	Arthritis, CDV prevention	NSAIDs *(Ibp, Cxb, Asc, Rxb, Nbm, Npx, Etd)*	600–1600 mg, 200 mg, 325 mg, 25 mg, 500 mg, 375 mg, 400 mg	NA	NA	0	Early/late	NA	NA	0.1	NA	Daily NSAID use was linked to increased crestal bone levels around hydroxyapatite‐coated single‐tooth implants after crown placement.
		68	265	ASA I–II	—	—	—	NA	2.26	Early/late	NA	NA	0.4		
Ursomanno et al. (2020)	RC	NA	1299	NA	PPIs	NA	NA	NA	5.5	NA	NA	NA	1.6 ± 3.5	NA	PPI medications are related to more loss of crestal bone at implant sites.
		NA	210	NA	—	—	—	NA	2.0	NA	NA	NA	1.0 ± 1.9		
Winnett et al. (2016)	RC	60	273	NA	NSAIDs (Ibp)	600 mg	4Xd 2 w	NA	44	Early/late	NA	NA	NA	NA	NSAIDS experienced 3.2 times more of radiographic bone loss greater than 30% of the vertical height and 1.9 times of failures.
		44	203	NA	—	—	—	NA	38	Early/late	NA	NA	NA		
Wu et al. (2014)	RC	50	94	ASA I–II	SSRIs	NA	NA	NA	10.6	Early/late	NA	NA	NA	NA	SSRI was associated with an increased risk of dental implants failure (HR: 6.28). Small implant diameters (≤ 4 mm) and smoking was also associated with higher risk of implant failure.
		440	882		—	—	—	NA	4.6	Early/late	NA	NA	NA		
Wu et al. (2017)	RC	58	133	ASA I–II	PPIs	NA	≥ 1 weeks	NA	6.8	Late	NA	NA	NA	NA	Subjects using PPIs had a higher risk of dental implant failure (HR: 2.73) compared to those who did not use the drugs. The use of NSAIDs was also associated with increased risk of dental implant failure (HR: 2.47).
		741	1640	ASA I–II	—	—	—	NA	3.2	Late	NA	NA	NA		
		NA	191	ASA I–II	NSAIDs	NA	≥ 1weeks	NA	6.2	Late	NA	NA	NA		
		NA	1582	ASA I–II	—	—	—	NA	3.2	Late	NA	NA	NA		
Wu et al. (2016)	RC	142	327	HTA	AHTNs	NA	NA	NA	0.6	Late	NA	NA	NA	NA	Treatment with antihypertensive drugs is associated with higher survival rate of osseointegrated implants.
		586	22	HTA	—	—	—	NA	4.7	Late	NA	NA	NA		
			1150	ASA I–II	—	—	—	NA	4.1	Late	NA	NA	NA		

Abbreviations: ACs, anticoagulants; AF/VF, Atrial/ventricular fibrillation; AHTNs, Antihypertensive Drugs/Agents; ASA, American Society of Anesthesiologists Classification; Asc, acetylsalicydic; BB, beta‐blockers; CC, Case control; CS, case series; CSECT, cross‐sectional; CT, concomitant connective tissue diseases; CXB, celecoxib; Db, Diabetes; DC, Depressive condition; Etd, etodolac; Flb, Flurbiprofen; GCA, giant cell arteritis; GD, Gastric disorders; HTA, hypertension; Ibp, ibuprofen; IHD ischemic heart disease; IMNs, Immunosuppressants agents; LE, systemic lupus erythematosus; LP, liquen planus; LT, liver transplant; MAOI monoamine oxidase Inhibitors; MG, myasthenia gravis; NA, not assessed; Nbm, nabumetone; Npx, naproxen; NSAIDs, Nonsteroidal Anti‐Inflammatory Drugs; OST, osteoporosis; OT, Organ transplant; PC, Prospective study; PN, polyarteritis nodosa; PPIs, Proton Pump Inhibitors; PSO, psoriasis; RA, Rheumatoid arthritis; RC, Retrospective Study; RCT, Randomized clinical trial; RXB, rofecoxib; SNRI, serotonin‐norepinephrine reuptake inhibitors; SS, Sjögren's syndrome; SSRIs, selective serotonin reuptake inhibitors; TCA, tricyclic antidepressants.

**TABLE 2:** Biological plausibility of medication‐related implant outcomes.

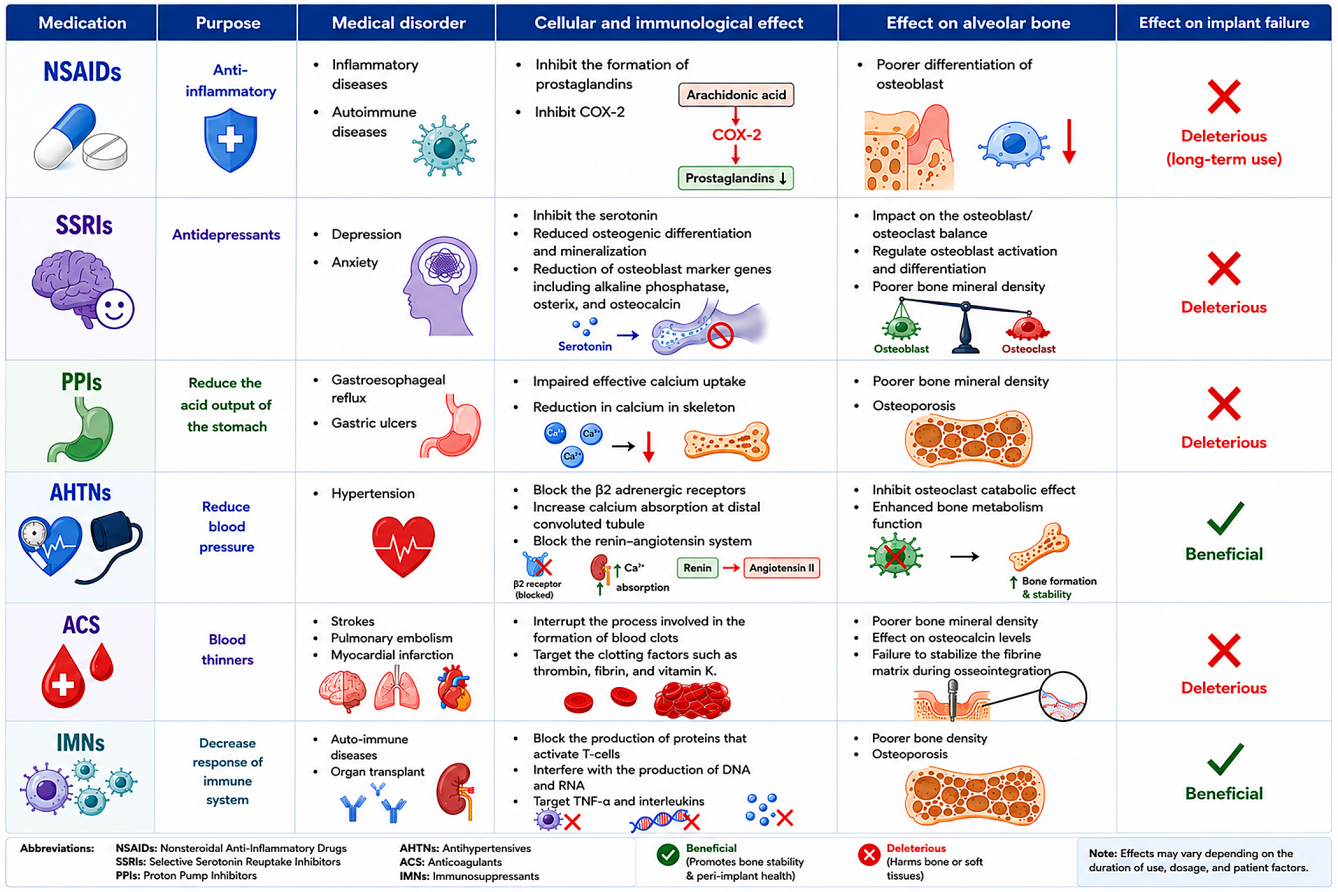

Concerning the medication intake, 6 groups were pooled as follows. Overall, 8 studies (Basson et al. 2023; Chrcanovic et al. 2016; Corbella et al. 2022; D'Orto et al. 2024; Masri et al. 2025; Seki et al. 2020; Tonini et al. 2022; Wu et al. 2016) reported the intake of AHTNs, 9 studies (Brizuela‐Velasco et al. 2021; Chatzopoulos and Wolff 2025a; Chaushu et al. 2023; D'Orto et al. 2024; Galletti et al. 2020; Gandhi and Bhatavdekar 2019; Jonas et al. 2024; Markovic et al. 2017; Romandini et al. 2021) reported the intake of ACs, 12 studies (Anitua et al. 2018; Basson et al. 2023; Chrcanovic et al. 2016, 2017a; El‐Sherbini 2018; Gu and Yu 2011; Hernandez et al. 2019; Khamis et al. 2019; Montebugnoli et al. 2015; Paredes et al. 2018; Petsinis et al. 2017; Radzewski and Osmola 2019) reported the intake of IMNs, 11 studies (Alissa et al. 2009; Basson et al. 2023; Jeffcoat et al. 1995; Kumchai et al. 2025; Reddy et al. 1990; Romandini et al. 2021; Sakka and Hanouneh 2013; M. Tattan et al. 2021; Urdaneta et al. 2011; Winnett et al. 2016; Wu et al. 2017) reported the intake of NSAIDs, 12 studies (Altay et al. 2019; Basson et al. 2023; Chatzopoulos and Wolff 2025b; Chrcanovic et al. 2016, 2017a; Corbella et al. 2022; Masri et al. 2023; Rogoszinski et al. 2022; Romandini et al. 2021; Ursomanno et al. 2020; Wu et al. 2017) reported the intake of PPIs, and 12 studies (Altay et al. 2018; Basson et al. 2023; Block and Mercante 2025; Carr et al. 2019; Chandra et al. 2021; Chrcanovic et al. 2017c; Corbella et al. 2022; Deepa et al. 2018; Hakam et al. 2021; Kotsailidi et al. 2023; Rodriguez‐Pena et al. 2022; Wu et al. 2014) reported the intake of SSRIs. Finally, 1 study reported pooled data regarding the use of PPIs, SSRIs, AHTNs and statins (M. Tattan et al. 2021).

### 3.2 Effect of AHTNs on Implant Therapy

Of the included studies, 8 evaluated the clinical outcomes associated with the concomitant use of dental implants and AHTNs (Basson et al. 2023; Chrcanovic et al. 2016; Corbella et al. 2022; D'Orto et al. 2024; Masri et al. 2025; Seki et al. 2020; Tonini et al. 2022; Wu et al. 2016). Particularly, 6 were retrospective studies (Chrcanovic et al. 2016; Corbella et al. 2022; Masri et al. 2025; Seki et al. 2020; Tonini et al. 2022; Wu et al. 2016) and 2 were case–control studies (Basson et al. 2023; D'Orto et al. 2024). In addition, 1 study evaluated data from patients taking AHTNs concomitantly with PPIs, SSRIs, and statins (Tattan et al. 2021).

With regard to early implant failure, 1 retrospective study (Masri et al. 2025) suggested that AHTNs may exert a protective effect on osseointegration. The overall early implant failure rate was 3.84% at the implant level and 13.6% at the patient level. Among patients receiving AHTNs, these rates decreased to 2.29% and 9.70%, respectively, with the differences reaching statistical significance (*p* = 0.059). Hence, the use of AHTNs was associated with a marginally significant reduced risk of early implant failure (OR = 0.618) (Masri et al. 2025). Supporting the potential protective effect of AHTNs, a lower rate of late implant failure (0.6% overall failure rate) was observed among AHTN users compared to nonusers, suggesting a possible beneficial influence on implant survival (OR = 0.14) (Wu et al. 2016). Notably, in the same study, untreated hypertensive patients demonstrated implant failure rates comparable to those of normotensive controls (Wu et al. 2016). In contrast, several studies consistently reported no significant association between AHTNs and implant failure. A case–control study that evaluated pooled data from patients taking AHTNs concomitantly with PPIs, SSRIs, and statins found no difference in early implant failure between patients receiving these medications and those who did not (Tattan et al. 2021). Moreover, a case–control study found no significant differences in early or late implant survival among patients using AHTNs alone or in combination with ACs (D'Orto et al. 2024). Focusing only on late implant failures, it was suggested no effect of AHTNs (Seki et al. 2020). Moreover, several reports identified, alike, no significant differences in implant survival between AHTN users and nonusers (Basson et al. 2023; Chrcanovic et al. 2016; Corbella et al. 2022; Tonini et al. 2022).

Concerning the occurrence of biological complications, an increased risk of peri‐implantitis (OR = 12.90) was reported in AHTN users, potentially linked to a higher incidence of soft tissue overgrowth, which may be associated with calcium channel blocker therapy (Seki et al. 2020). Mean marginal bone loss was also higher in the AHTNs than in the non‐AHTNs group (Seki et al. 2020). Contrarily, no significant association between AHTNs use and marginal bone loss or other peri‐implant conditions was reported in another study (D'Orto et al. 2024).

### 3.3 Effect of ACs on Implant Therapy

Of the included studies, 9 evaluated the clinical outcomes related to the use of ACs and implant therapy (Brizuela‐Velasco et al. 2021; Chatzopoulos and Wolff 2025a; Chaushu et al. 2023; D'Orto et al. 2024; Galletti et al. 2020; Gandhi and Bhatavdekar 2019; Jonas et al. 2024; Markovic et al. 2017; Romandini et al. 2021). Specifically, 5 were retrospective studies (Brizuela‐Velasco et al. 2021; Chatzopoulos and Wolff 2025a; Chaushu et al. 2023; Galletti et al. 2020; Jonas et al. 2024), 1 was a case–control study (D'Orto et al. 2024), 1 was a case series (Gandhi and Bhatavdekar 2019), 1 was cross‐sectional (Romandini et al. 2021), and 1 was an RCT (Markovic et al. 2017).

Regarding early implant failure, all the included studies reported relevant outcomes. In total, 3 studies specifically evaluated only early failure (Chaushu et al. 2023; Gandhi and Bhatavdekar 2019; Jonas et al. 2024). A retrospective cohort study evaluating the impact of antiplatelet and AC therapy on early implant failure following sinus floor augmentation found no statistically significant association between AC use and early failure (Jonas et al. 2024). Nonetheless, at the patient level, the failure rate among AC users was nearly twice as high (36.4%) as that of healthy individuals (18.2%), although this difference did not reach statistical significance (Jonas et al. 2024). Consistent with these findings, another study published elsewhere also found no significant association between AC use and early implant failure at the patient level (Chaushu et al. 2023). However, they did report a statistically significant association at the implant level, with AC use doubling the risk of implant failure (Chaushu et al. 2023). Conversely, in a case series examining flapless dental implant placement in patients undergoing uninterrupted direct AC therapy, no early implant failures were observed (Gandhi and Bhatavdekar 2019).

Regarding the association between either early or late failure, four studies found no significant association between AC use and early or late implant failure (Chatzopoulos and Wolff 2025b; D'Orto et al. 2024; Galletti et al. 2020; Markovic et al. 2017). No association was found between early or late implant survival and the use of ACs alone or in combination with AHTN medications (D'Orto et al. 2024). In contrast to these findings, one study reported a significant association between AC use and early and late implant failure (Brizuela‐Velasco et al. 2021). This retrospective study evaluated 110 patients on AC who demonstrated a substantially increased risk of implant loss (OR = 28.2) (Brizuela‐Velasco et al. 2021). It should be noted, however, that this study included elderly patients diagnosed with systemic diseases (Brizuela‐Velasco et al. 2021). Therefore, age and comorbidities may represent determinants of implant outcomes other than the intake of ACs.

In relation to biological complications, only 1 study reported that the use of ACs may exert a protective effect against the development of peri‐implantitis (OR = 0.08). The authors attributed this finding to the potential secondary anti‐inflammatory effects of ACs (Romandini et al. 2021).

### 3.4 Effect of IMNs on Implant Therapy

A total of 12 articles evaluated the use of dental implants in patients undergoing IMN therapy. Of these, 4 were retrospective studies (Anitua et al. 2018; Chrcanovic et al. 2016, 2017a; Petsinis et al. 2017), 5 were prospective studies (Hernandez et al. 2019; Khamis et al. 2019; Montebugnoli et al. 2015; Paredes et al. 2018; Radzewski and Osmola 2019), two were case–control studies (Basson et al. 2023; El‐Sherbini 2018), and 1 was a case series (Gu and Yu 2011). Regarding the underlying systemic condition, 2 studies (Anitua et al. 2018; Khamis et al. 2019) evaluated implant outcomes in patients with lichen planus receiving systemic IMNs. In 5 studies, IMNs were associated with organ transplantation. In 2 other studies, the use of IMNs was administered for the treatment of autoimmune diseases (El‐Sherbini 2018; Petsinis et al. 2017). Conversely, in 3 studies, the specific indication for IMNs was not reported (Basson et al. 2023; Chrcanovic et al. 2016, 2017a).

Regarding implant‐level failure rate, a low late failure rate (1.5%) was observed in patients taking systemic glucocorticoid therapy (Anitua et al. 2018). No significant effect of systemic glucocorticoids on implant failure (2.7%) over a 3‐year follow‐up was noticed in another study on patients with rheumatoid arthritis. Early bone loss, however, was significantly higher (14.3%), attributable to a single prosthesis supported by multiple dental implants (Petsinis et al. 2017). Another report showed an implant failure rate relatively high (13.5%), although it did not reach statistical significance in terms of overall implant failure when compared to non‐IMN users (Chrcanovic et al. 2016). The other 2 studies did not assess the therapeutic outcomes at the implant level (Basson et al. 2023; Chrcanovic et al. 2017a). The remaining studies reported no implant failures, supporting the safety of implant therapy under IMNs in both the short and long term (El‐Sherbini 2018; Gu and Yu 2011; Khamis et al. 2019; Montebugnoli et al. 2015; Paredes et al. 2018; Radzewski and Osmola 2019).

In total, 2 studies (Hernandez et al. 2019; Paredes et al. 2018) reported the prevalence of peri‐implant mucositis and peri‐implantitis at the implant level with a long‐term follow‐up in patients taking IMNs. Mucositis ranged from 35.4% to 46.8% and peri‐implantitis from 4.2% to 5.1% (Hernandez et al. 2019; Paredes et al. 2018). Hence, there was no suggestion of an association between the intake of IMNs and biological complications. Regarding marginal bone loss, only 1 of the included studies documented statistically significant differences in a 4‐year follow‐up (Khamis et al. 2019). Notably, the group stopping IMNs 12 weeks after implant placement exhibited greater marginal bone loss compared to both the long‐term intake of IMNs patients and non‐IMNs patients (Khamis et al. 2019).

### 3.5 Effect of NSAIDS on Implant Therapy

Of the included studies, 8 evaluated the influence of NSAID therapy on implant outcomes (Alissa et al. 2009; Basson et al. 2023; Jeffcoat et al. 1995; Kumchai et al. 2025; Reddy et al. 1990; Sakka and Hanouneh 2013; Tattan et al. 2021; Urdaneta et al. 2011; Winnett et al. 2016; Wu et al. 2014). Among these, 5 were RCTs (Alissa et al. 2009; Jeffcoat et al. 1995; Kumchai et al. 2025; Reddy et al. 1990; Sakka and Hanouneh 2013), 3 were retrospective studies (Urdaneta et al. 2011; Winnett et al. 2016; Wu et al. 2016), and 2 were case–control studies (Basson et al. 2023; Tattan et al. 2021).

Regarding early implant failure, an RCT yielded no differences between patients who received implant therapy with or without a 1‐week (short‐term) post‐operative course of NSAIDs administered four times daily (Alissa et al. 2009). Similarly, the case–control study reported no significant difference in early implant failures (OR = 1.06) (Basson et al. 2023). Notably, there was also no short‐term difference in implant failure between groups receiving NSAIDs and placebo. However, when patients with implant failure were retrospectively surveyed about their NSAIDs use, a higher proportion (67.7%) reported NSAIDs consumption compared to the general population (26.1%), as indicated by the National Health and Nutrition Examination Survey (Kumchai et al. 2025). Supporting this observation, other studies associated long‐term NSAIDs use with a higher risk of implant failure (HR = 2.47) (Wu et al. 2017), and nearly twice the number of failures (Winnett et al. 2016).

None of the studies reported the prevalence of peri‐implant diseases, whereas 3 studies did report on marginal bone level values (Alissa et al. 2009; Jeffcoat et al. 1995; Kumchai et al. 2025). A RCT found no significant differences in marginal bone levels between patients who received a 1‐week postoperative NSAID regimen and those who did not (Alissa et al. 2009). A similar result was observed in a more recent RCT, where marginal bone loss over 16 weeks did not differ significantly between NSAIDs and placebo groups (Kumchai et al. 2025). In contrast, another RCT reported reduced bone loss in implants where NSAIDs were prescribed for 3 months compared to a placebo (Jeffcoat et al. 1995). Although specific values were not provided, additional studies indicated that NSAID use was associated with increased bone density during the early healing phase (Reddy et al. 1990), higher crestal bone levels (Urdaneta et al. 2011), or, conversely, a 3.2‐fold increase in cases with radiographic bone loss exceeding 30% of the vertical implant height (Winnett et al. 2016). Although osteoblasts have the capacity to produce prostaglandins, where PGE2 is dominant, through the COX pathway, the evidence linking this to peri‐implant bone gain or bone stability is yet inconclusive.

### 3.6 Effect of PPIs on Implant Therapy

Among the included studies, 9 had a retrospective design and evaluated therapeutic outcomes (Chatzopoulos and Wolff 2025b; Chaushu et al. 2023; Chrcanovic et al. 2016, 2017a, 2017b; Corbella et al. 2022; Masri et al. 2023; Rodriguez‐Pena et al. 2022; Rogoszinski et al. 2022; Ursomanno et al. 2020; Wu et al. 2017).

Regarding implant failure, 1 retrospective study reported a significantly increased risk in patients using PPIs with an OR = 1.40. Notably, omeprazole use was associated with a higher risk of implant failure with an HR of 1.45 compared to nonusers (Chatzopoulos and Wolff 2025b). A series of studies from the same group demonstrated a negative impact of PPI use on implant survival. These included early implant failure rates of 2.4% and late failure rates of 9.6% of PPI users. The authors reported a significantly reduced implant survival rate associated with PPI use (HR = 2.81) (95% CI: 1.139, 6.937), and a significant difference in implant survival between PPI users and nonusers (OR: 2.9). Furthermore, among patients receiving more than three implants, PPI use was linked to a higher risk of failure (OR = 1.8) (95% CI: 1.105, 3.233) (Chrcanovic et al. 2016, 2017a, 2017b).

Similarly, another study found a significantly increased risk of early implant failure associated with PPI use (OR: 1.91) (Masri et al. 2025). Others also reported a higher implant failure risk in PPI users (HR = 2.73) (Wu et al. 2017). In contrast, after adjusting for confounding factors, another study found no significant association between PPI use and implant failure (OR = 0.8) (95% CI: 0.56–1.15) (Rogoszinski et al. 2022).

With respect to other implant‐related parameters, only 1 study reported on marginal bone levels, indicating greater crestal bone loss in PPI users (Ursomanno et al. 2020). Another study evaluating peri‐implantitis found no significant association between PPI use and disease prevalence (Rogoszinski et al. 2022).

### 3.7 Effect of SSRIs on Implant Therapy

A total of 13 studies investigated therapy outcomes. These included 11 RCTs (Altay et al. 2018; Block and Mercante 2025; Carr et al. 2019; Chandra et al. 2021; Chrcanovic et al. 2017c; Corbella et al. 2022; Deepa et al. 2018; Hakam et al. 2021; Kotsailidi et al. 2023; Rodriguez‐Pena et al. 2022; Wu et al. 2014), and 2 case–control studies (Basson et al. 2023; Tattan et al. 2021).

Regarding implant failure, 1 RCT reported a higher incidence in SSRI users (OR = 3.12). However, the result did not reach statistical significance (Altay et al. 2018). A similar trend was observed by others, where the relative risk for implant failure in SSRI users was 2.8 (Block and Mercante 2025). Another RCT also reported a higher failure rate among SSRI users (12%) compared to ASA I–II subjects (5.8%) (Chandra et al. 2021). Consistent findings were also noted in another RCT (Chrcanovic et al. 2017c) and by additional studies, which found a statistically significant increase in implant failure rates among SSRI users, particularly among patients over 50 years of age (Deepa et al. 2018), with reported HR and ORs as high as 4.53 (Rodriguez‐Pena et al. 2022) and 6.8 (Wu et al. 2014), respectively. In contrast, 4 studies found no significant difference in implant failure rates between SSRI users and nonusers (Basson et al. 2023; Carr et al. 2019; Corbella et al. 2022; Hakam et al. 2021), or when a variety of medications were combined (Tattan et al. 2021).

Only one study reported on marginal bone levels, noting significantly greater bone loss in SSRI users after a mean functional period of 3.8 years (Kotsailidi et al. 2023). However, none of the included studies provided data on the prevalence of peri‐implant diseases.

### 3.8 Risk of Bias Assessment

Appendices S2 and S3 report the risk of bias for nonrandomized and for randomized studies, respectively.

## 4 Discussion

### 4.1 Principal Findings

The aim of this scoping review was to synthesize the available evidence on the influence of commonly prescribed systemic medications, such as AHTNs, ACs, IMNs, NSAIDs, PPIs, and SSRIs, on implant‐related outcomes. Overall, the findings unveiled a complex and heterogeneous body of evidence, often limited by the lack of well‐designed, high‐quality studies. While certain medications, such as ACs, long‐term NSAIDs use, prolonged PPI therapy, and SSRIs in some studies were associated with increased risks of implant failure or marginal bone loss, other studies reported no clinically significant effects. In contrast, short‐term NSAID use did not appear to adversely affect implant survival or marginal bone levels. Interestingly, in some studies, the use of AHTNs and IMNs was associated with a potentially positive effect on implant outcomes.

### 4.2 Biological Plausibility of Our Findings

In general lines, the intake of AHTNs was suggested not to be deleterious. Instead, few studies identified this medication as protective (or no effect) on implant failure. The potential mechanisms by which AHTNs may influence bone metabolism involve several distinct pathways. First, beta‐blockers, widely used for the management of hypertension, reduce blood pressure by inhibiting β‐adrenergic receptors. These receptors, particularly β2‐receptors, are expressed on osteoblasts and osteoclasts, where their activation promotes bone resorption and decreases bone formation. Thus, β‐blockade may have a protective effect on bone by attenuating sympathetic‐induced bone loss (Ducy et al. 2000; Moore et al. 1993). Second, thiazide diuretics act by inhibiting the thiazide‐sensitive sodium‐chloride cotransporter (NCC) in the renal distal tubules, reducing urinary calcium excretion and enhancing calcium reabsorption (Bazzini et al. 2005). This leads to elevated serum calcium levels, which suppress parathyroid hormone (PTH) secretion, thereby decreasing bone turnover and potentially increasing bone mineral density (BMD) (Bolland et al. 2007). Furthermore, thiazides may directly stimulate osteoblastic proliferation and differentiation (Aubin et al. 1996).

Third, angiotensin‐converting enzyme (ACE) inhibitors and angiotensin II receptor blockers (ARBs) modulate the renin‐angiotensin‐aldosterone system (RAAS), which is active both systemically and locally in bone tissue (Nakagami et al. 2013). Inhibition of the RAAS may positively influence bone metabolism, as ACE inhibitors and ARBs have been associated with reduced fracture risk and enhanced BMD (Rejnmark et al. 2006) Additionally, ARBs may suppress bone resorption by inhibiting tartrate‐resistant acid phosphatase, an enzyme involved in osteoclastic activity (Shimizu et al. 2008). Preclinical studies further support the beneficial skeletal effects of RAAS inhibition. Animal models have demonstrated that losartan, an ARB, improves bone quality and reverses osteoporotic changes following ovariectomy‐induced bone loss (Abuohashish et al. 2017; Donmez et al. 2012). Similarly, ACE inhibitors such as enalapril and captopril have been shown to prevent bone loss in diabetic and osteoporotic animal models (Asaba et al. 2009; Diao et al. 2014). Despite the biological rationale behind the positive effect of AHTNs on implant outcomes, several clinical investigations have not observed significant differences (Basson et al. 2023; Chrcanovic et al. 2016; Corbella et al. 2022; Tonini et al. 2022). In line with these findings, a clinical study that evaluated structural and histological parameters of alveolar bone samples collected from hypertensive patients treated with renin‐angiotensin system antagonists, as well as from normotensive individuals, demonstrated that patients receiving RAS antagonists exhibited alveolar bone with preserved structure and normal histological characteristics (Fabris et al. 2017). Considering the systemic anabolic effects of AHTN medications on bone metabolism, beneficial effects on alveolar bone cannot be excluded (Abuohashish et al. 2017; Asaba et al. 2009; Aubin et al. 1996; Bazzini et al. 2005; Bolland et al. 2007; Diao et al. 2014; Donmez et al. 2012; Ducy et al. 2000; Moore et al. 1993; Nakagami et al. 2013; Shimizu et al. 2008). However, current clinical evidence does not consistently demonstrate that AHTN's drugs exert a protective effect on osseointegration or positively influence dental implant survival. In addition, beta‐blocker therapy may be associated with an increased incidence of gingival hyperplasia, potentially elevating the risk for peri‐implant diseases (Seki et al. 2020).

The potential impact of ACs on dental implant survival remains a topic of ongoing debate. Some evidence suggests that AC agents may affect bone metabolism through various mechanisms (Caraballo et al. 1999; Gigi et al. 2012). ACs can be broadly classified into two main categories: direct oral ACs (DOACs) and vitamin K antagonists (VKAs). Regarding DOACs, Gigi and coworkers reported that rivaroxaban may influence osteoblastic cell proliferation and modulate hormonal pathways, potentially impairing early osseointegration, although osteoblastic mineralization appeared to remain unaffected (Gigi et al. 2012). Conversely, a clinical study has found no detrimental effects of rivaroxaban on osseointegration (Galletti et al. 2020). Vitamin K antagonists, such as warfarin, may interfere with the function of vitamin K‐dependent proteins involved in bone metabolism, including osteocalcin, which is critical for bone matrix mineralization. As vitamin K is required for osteocalcin's posttranslational γ‐carboxylation, inhibition by VKAs may impair osteoblastic function and bone quality. Clinical studies have linked warfarin use to reduced bone mineral density (BMD) and increased fracture risk (Caraballo et al. 1999; Pilkey et al. 2007).

In addition to DOACs and VKAs, prolonged heparin therapy, particularly with unfractionated heparin (UFH), has been associated with adverse effects on bone health, leading to decreased BMD and increased fracture risk (Monreal et al. 1994). Although low molecular weight heparins (LMWHs) may present a lower risk profile, they are not entirely without bone‐related side effects. Overall, the current evidence regarding the effects of ACs on bone homeostasis and implant outcomes remains conflicting and inconclusive. Although ACs do not appear to contraindicate implant placement, caution is warranted during the early healing phase, particularly in high‐risk individuals (Bacci et al. 2011). This is especially relevant due to the increased risk of intraoperative and postoperative bleeding, which may contribute to complications such as hematoma formation, delayed wound healing, and an elevated risk of infection (Jonas et al. 2024). In summary, the current evidence on the effects of ACs on implant outcomes remains limited and somewhat contradictory. While ACs do not appear to contraindicate implant placement, caution is advised during early healing. Patient‐specific factors such as age and comorbidities may play a more significant role. Further high‐quality studies are needed to clarify these associations.

In general, the potential impact of IMNs on dental implant treatment may be linked to impaired inflammatory response, altered bone remodeling, and immune system dysfunction (Burtscher and Dalla Torre 2022; Duttenhoefer et al. 2019). In the early stages following implant placement, IMNs may significantly impair peri‐implant bone healing and osseointegration. Several preclinical animal studies have demonstrated a reduction in bone density surrounding implants under immunosuppressive conditions (de Molon et al. 2017; El Hadary et al. 2011). Commonly prescribed IMNs such as cyclosporine A, glucocorticoids, and methotrexate have been shown to adversely affect the osteogenic process by inhibiting osteoblast proliferation, differentiation, and activity while simultaneously decreasing bone mass and increasing osteoblastic apoptosis (Chen et al. 2025; Fu et al. 2012). These biological alterations may compromise osseointegration and increase the risk of early implant failure. Glucocorticoids are among the most commonly used for the treatment of autoimmune diseases. Despite the adverse effects, dental implant therapy is generally considered predictable and is associated with a high implant survival rate and comparable with healthy patients. However, when implant loss does occur, it typically happens before implant loading (Alenazi 2021; Hyldahl et al. 2025). Moreover, an altered immune system caused by IMNs may result in a reduced ability to respond effectively to bacterial challenge in the oral environment, reducing the immune cells' activity like T‐cells and macrophages (Aldabeab Khalid Eisa 2024; Bornstein et al. 2009; Duttenhoefer et al. 2019). Therefore, long‐term therapy may lead to an increased risk of infection, which is considered one of its potential side effects (Henrickson et al. 2016). IMNs may exert adverse effects during the early stages of implant therapy, particularly in relation to osseointegration, as demonstrated predominantly in preclinical models. Despite the limited and heterogeneous nature of available clinical studies in humans, evidence indicates that IMNs do not significantly compromise implant survival, nor do they appear to increase the prevalence of peri‐implant mucositis or peri‐implantitis.

NSAIDs are among the most commonly prescribed analgesics worldwide and represent the first‐line option for managing pain and inflammation following a variety of oral surgical procedures, including dental implant placement (Kotowska‐Rodziewicz et al. 2023). Their prescription varies depending on factors such as the active compound, the patient's systemic condition, or other medications that could negatively interact with NSAIDs, the extent of the surgical intervention (e.g., implant placement with or without bone augmentation), and clinician preference. In our review, while short‐term NSAIDs use during the post‐operative period appears to have no significant adverse effect on implant‐related outcomes, prolonged use seems to be associated with an increased risk of implant failure and marginal bone loss. These findings are similar to the ones reported in previous reviews on this topic (Gomes et al. 2015; Kalyvas and Tarenidou 2008). The biological plausibility behind these findings may lie in the mechanism of action of NSAIDs, which exert their effects by inhibiting cyclooxygenase (COX) enzymes, particularly COX‐2, essential for modulating inflammation and bone remodeling. However, most NSAIDs also inhibit COX‐1, a constitutive enzyme involved in maintaining gastrointestinal integrity and contributing to bone hemostasis (Kotowska‐Rodziewicz et al. 2023; van Esch et al. 2013). This nonselective inhibition may inadvertently disrupt the delicate balance of bone metabolism, particularly through reduced levels of prostaglandin E2 (PGE2), a key mediator of osteoblast and osteoclast activity. Moreover, NSAIDs may alter the RANKL/OPG ratio and trigger systemic inflammatory responses, further impairing bone healing. Preclinical and in vitro studies support these concerns, highlighting a potential link between NSAID use and impaired osseointegration (van Esch et al. 2013). Thus, while NSAIDs are valuable for short‐term analgesia without compromising implant therapy outcomes, their long‐term use should be approached with caution.

PPIs are widely prescribed medications primarily used to suppress gastric acid secretion in the treatment of gastroesophageal reflux disease, peptic ulcers, and related conditions. A recent systematic review examining global prescribing trends reported that approximately 25% of adults use PPIs, with the majority being under 65 years of age (63%), predominantly female (56%), and mainly of white ethnicity (75%) (Shanika et al. 2023). Notably, nearly two‐thirds of these individuals are prescribed high doses, while 25% continue therapy for over one year, and 28% of these long‐term users remain on PPIs for more than three years (Shanika et al. 2023). Although short‐term PPI use is generally regarded as safe, accumulating evidence links prolonged use to adverse effects, including increased risk of bone fractures and impaired absorption of essential vitamins and minerals (Shanika et al. 2023). The biological rationale behind these effects centers on the suppression of gastric acid, which is crucial for optimal calcium absorption. Reduced gastric acidity can lead to diminished systemic calcium availability, a mineral integral to bone remodeling and mineralization processes (Kopic and Geibel 2010, 2013). Insufficient calcium disrupts the delicate balance between osteoclastic bone resorption and osteoblastic formation, potentially resulting in decreased bone mineral density (Yeum et al. 2025). Furthermore, PPI‐induced hypomagnesemia may exacerbate this imbalance by impairing osteoblast and osteoclast function, interfering with the hydroxylation of vitamin D metabolites, and inducing target organ resistance to parathyroid hormone (Briganti et al. 2021). These combined effects may compromise the alveolar bone, thereby increasing the risk of implant failure in patients undergoing dental implant therapy.

SSRIs are among the most commonly prescribed medications for the treatment of depression and other psychiatric conditions, such as generalized anxiety disorder, bulimia nervosa, and panic disorders, due to their established safety profile, efficacy, and overall tolerability (Chu and Wadhwa 2025). According to a recent global market analysis, the antidepressant drug market was valued at approximately USD 18.7 billion in 2024 and is projected to grow at a compound annual growth rate of 7% between 2025 and 2034. This growth is largely driven by increased awareness of mental health disorders, the demand for effective pharmacological therapies, and the expanded accessibility of generic SSRIs, which have significantly lowered treatment costs and increased availability across diverse populations (Global Market Insights 2025). In this review, an emerging body of evidence suggests that SSRIs may be associated with early and late implant failure (Altay et al. 2018; Block and Mercante 2025; Chandra et al. 2021; Chrcanovic et al. 2017c; Deepa et al. 2018; Kotsailidi et al. 2023; Rodriguez‐Pena et al. 2022; Wu et al. 2014). However, only 1 study assessed changes in marginal bone levels, reporting significantly greater bone loss among SSRI users compared to nonusers (Kotsailidi et al. 2023), and no study to date has reported direct associations between SSRIs and peri‐implant diseases. These findings align with a recent evidence synthesis by the Evidence Synthesis Program Coordinating Center from the VHA Office of Dentistry, Oral Health Quality Group, which concluded that there is moderate‐strength evidence linking SSRI use with increased risk of implant failure, while evidence regarding their influence on peri‐implant disease remains inconclusive (Parr and Beech 2022). The potential impact of SSRIs on implant osseointegration and marginal bone stability can be explained by several biologically plausible mechanisms. SSRIs exert their primary pharmacological action by increasing serotonin levels in the central nervous system through inhibition of its reuptake. However, serotonin also plays a regulatory role in bone metabolism. Specifically, peripheral serotonin has been shown to inhibit osteoblast proliferation through activation of 5‐HT1B receptors, thereby impairing new bone formation (Yadav et al. 2008). Elevated extracellular serotonin levels induced by SSRI use may therefore affect the bone remodeling process necessary for successful osseointegration. In addition, SSRIs have been associated with increased osteoclast activity, by a reduction in genes like alkaline phosphatase, osteocalcin, and osterix, and reductions in bone mineral density, further shifting the balance toward bone resorption (Maes et al. 1998; Warden et al. 2005). These effects, combined with SSRI‐induced modulation of inflammatory mediators such as tumor necrosis factor‐alpha (TNF‐α) and interleukin‐6 (IL‐6), may create a local pro‐inflammatory environment that predisposes to peri‐implant bone loss (Maes et al. 1998). Collectively, these mechanisms provide a compelling biological rationale for the observed clinical associations between SSRI use and compromised implant therapeutic outcomes.

Increased life expectancy increases the prevalence of polypharmacy. Drug interactions in patients taking multiple medications for different disorders may lead to unexpected reactions and produce synergistic or antagonistic effects. A recent retrospective study unveiled the drug–drug interactions in patients demanding dental treatment. Interestingly, key high‐risk interactions included epinephrine with beta‐blockers, with patients aged 31–60 years being the ones who experienced the most adverse events (Colibasanu et al. 2025). No adverse events were reported in patients taking multiple drugs in any of the included studies. However, the concerted action of multiple medication intake on early and late implant outcomes must be explored in future trials.

### 4.3 Limitations and Future Outlook

Shortcomings associated with the methodology of this review and the included studies must be disclosed. The objective of this scoping review was to update and broaden the scope of a systematic review conducted by our group nearly a decade ago (Chappuis et al. 2018). Overall, 17 studies were included in the synthesis, while the current review resulted in 51 eligible studies. However, due to the heterogeneity exhibited by the included studies, meta‐analysis was not feasible. Accordingly, the authors opted for a qualitative assessment. In this sense, it is relevant to notice that despite the solidness being concluded concerning the association of medication intake and implant failure, data associating them with biological complications is still sparse. Hence, long‐term outcomes reporting the prevalence of mucositis and peri‐implantitis are encouraged to understand the role of medication intake and peri‐implant tissue stability. As highlighted in the previous review (Chappuis et al. 2018), the effect of poly‐medication is underreported, and the synergistic effects of medications and their effect on implant outcomes are to be explored. In this sense, it is important to remark that other confounders known to affect early and late outcomes, namely surgical‐ and prosthetic‐related factors or patient risk profile and compliance with supportive care, amongst many others, must be explored in multivariate analyses to provide adjusted odds to better explore the effect of medication intake on implant‐related outcomes.

## 5 Conclusion

In the planning of implant treatment, the clinician is recommended to pay particular attention to patients on ACs, long‐term NSAIDs use, prolonged use of PPIs, and SSRIs, since they may be at increased risks of implant failure and/or marginal bone loss. However, the association between biological implant complications and systemic medication intake remains underreported. These mixed findings, along with the potential impact of comorbidities, underscore the need for cautious interpretation and further investigation. In addition, it is recommended prospectively and meticulously to document medication in implant patients to further strengthen future meta‐analyses clinical recommendations.

## Author Contributions

**Alberto Monje:** conceptualization, writing – original draft. **Maria Costanza Soldini:** methodology. **Emilio Couso‐Queiruga:** methodology. **Vivianne Chappuis:** conceptualization, validation. **Álvaro Babiano:** methodology.

## Conflicts of Interest

The authors declare no conflicts of interest.

## Supporting information


**Table S1:** Search strategy for MEDLINE (PubMed) until 11th June 2025.
**Table S2:** Risk Of Bias In Nonrandomized Studies—of Interventions, Version 2 (ROBINS‐I).
**Table S3:** Risk‐of‐bias tool for randomized trials (RoB 2).

**Data S1:** Preferred Reporting Items for Systematic reviews and Meta‐Analyses extension for Scoping Reviews (PRISMA‐ScR) Checklist.

## Data Availability

The data that support the findings of this study are available on request from the corresponding author. The data are not publicly available due to privacy or ethical restrictions.
