# Supplementary material for: Effect of Medication Intake on Dental Implant‐Related Outcomes—A Scoping Review
Source: Clin Oral Implants Res. 2026 Jul 5;37(9):1033–56. doi: 10.1111/clr.70151 (PMC13542757; doi:10.1111/clr.70151)
Supplement: Supplementary file 1 [file CLR-37-1033-s001.docx]

**Supplementary table 1.** Search strategy for MEDLINE (PubMed) until 11^th^ June 2025

| 1 | (("dental implants" OR "dental implantation, endosseous"[MeSH Terms] OR (("dental implantation, endosseous") AND "abnormalities, drug-induced"[MeSH Terms]) OR serotonin uptake inhibitors [MeSH Terms] OR "anti-inflammatory agents, non-steroidal"[MeSH Terms] OR "adrenergic beta-antagonists"[MeSH Terms] OR "angiotensin-converting enzyme inhibitors"[MeSH Terms] OR "proton pump inhibitors"[MeSH Terms] OR "Immunosuppressive Agents"[MeSH Terms] OR "immunosuppressive drugs" OR "immunosuppression" OR "immunosuppressive therapy" OR "immunosuppressants") AND "survival analysis"[MeSH Terms] AND (Classical Article[ptyp] OR Clinical Study[ptyp] OR Clinical Trial[ptyp]) AND "humans"[MeSH Terms]) |
| --- | --- |
| 2 | ("Dental Implants"[Mesh] OR "dental implant placement" OR "implant surgery" OR "oral implantology") AND ( "Medically Compromised Patients" OR "systemic disease" OR "chronic disease" OR diabetes OR osteoporosis OR "cardiovascular disease" OR "renal disease" OR hypertension OR"Concomitant medication" OR corticosteroids OR immunosuppressants OR anticoagulants OR "non-steroidal anti-inflammatory drugs" OR NSAIDs OR selective serotonin reuptake inhibitors OR SSRIs OR "proton pump inhibitors" OR PPIs OR "antihypertensive drugs" OR "antihypertensive agents" OR "immunosuppressive drugs" OR "immunosuppression" OR "immunosuppressive therapy" OR "immunosuppressants") AND ( "Treatment Outcome"[Mesh] OR "implant survival" OR "implant failure" OR osseointegration OR "bone loss" OR "peri-implantitis" OR "peri-implant mucositis" OR "biological complication*" OR "implant complications" OR "marginal bone loss" ) |
| 3 | ((((medication-related[Title/Abstract] OR drug-related[Title/Abstract]) OR bisphosphonate-related[Title/Abstract]) AND dental implant failure[Title/Abstract]) OR dental im- plant survival[Title/Abstract]) OR (("dental implants"[MeSH Terms] OR ("dental"[All Fields] AND "implants"[All Fields]) OR "dental implants"[All Fields] OR ("dental"[All Fields] AND "implant"[All Fields]) OR "dental implant"[All Fields]) AND biological complication[Title/Abstract]) OR (("dental implants"[MeSH Terms] OR ("dental"[All Fields] AND "implants"[All Fields]) OR "dental implants"[All Fields] OR ("dental"[All Fields] AND "implant"[All Fields]) OR "dental implant"[All Fields]) AND technical complication[Title/Abstract]) |
| 4 | #1 #2 #3 Filters: English, Humans |
| 5 | ("Dental Implants"[MeSH Terms] OR "dental implant"[All Fields] OR "implant survival"[All Fields] OR "implant failure"[All Fields] OR "peri-implantitis"[All Fields] OR "peri-implant disease"[All Fields] OR "peri-implant mucositis"[All Fields] OR "bone loss"[All Fields] OR "marginal bone loss"[All Fields]) AND ("Anticoagulants"[MeSH Terms] OR "anticoagulant agents"[All Fields] OR "blood thinners"[All Fields] OR "warfarin"[All Fields] OR "coumadin"[All Fields] OR "apixaban"[All Fields] OR "rivaroxaban"[All Fields] OR "dabigatran"[All Fields] OR "edoxaban"[All Fields] OR "heparin"[All Fields] OR "low molecular weight heparin"[All Fields]) |
| 6 | ("Dental Implants"[MeSH Terms] OR "dental implant"[All Fields] OR "implant survival"[All Fields] OR "implant failure"[All Fields] OR "peri-implantitis"[All Fields] OR "peri-implant disease"[All Fields] OR "peri-implant mucositis"[All Fields] OR "bone loss"[All Fields] OR "marginal bone loss"[All Fields]) AND ("Antihypertensive Agents"[MeSH Terms] OR "antihypertensive drugs"[All Fields] OR "antihypertensive medication"[All Fields] OR "Angiotensin-Converting Enzyme Inhibitors"[MeSH Terms] OR "ACE inhibitors"[All Fields] OR "Angiotensin Receptor Antagonists"[MeSH Terms] OR "ARBs"[All Fields] OR "Adrenergic beta-Antagonists"[MeSH Terms] OR "beta blockers"[All Fields] OR "Calcium Channel Blockers"[MeSH Terms] OR "calcium antagonists"[All Fields] OR "Diuretics"[MeSH Terms] OR "thiazides"[All Fields] OR "loop diuretics"[All Fields]) |
| 7 | ("Dental Implants"[MeSH Terms] OR "dental implant"[All Fields] OR "implant survival"[All Fields] OR "implant failure"[All Fields] OR "peri-implantitis"[All Fields] OR "peri-implant disease"[All Fields] OR "peri-implant mucositis"[All Fields] OR "bone loss"[All Fields] OR "marginal bone loss"[All Fields]) AND ("Immunosuppressive Agents"[MeSH Terms] OR "immunosuppressive drugs"[All Fields] OR "immunosuppressive therapy"[All Fields] OR "immunosuppression"[All Fields] OR "immunosuppressants"[All Fields] OR "Organ Transplantation"[MeSH Terms] OR "Transplant Recipients"[MeSH Terms] OR "Autoimmune Diseases"[MeSH Terms] OR "Glucocorticoids"[MeSH Terms] OR "Corticosteroids"[All Fields] OR "Methotrexate"[All Fields] OR "Cyclosporine"[All Fields] OR "Tacrolimus"[All Fields] OR "Azathioprine"[All Fields] OR "Mycophenolate mofetil"[All Fields]) |
| 8 | ("Dental Implants"[MeSH Terms] OR "dental implant"[All Fields] OR "implant survival"[All Fields] OR "implant failure"[All Fields] OR "peri-implantitis"[All Fields] OR "peri-implant disease"[All Fields] OR "peri-implant mucositis"[All Fields] OR "bone loss"[All Fields] OR "marginal bone loss"[All Fields]) AND ("anti inflammatory agents, non steroidal"[MeSH Terms] OR "NSAIDs"[All Fields] OR "non-steroidal anti-inflammatory drugs"[All Fields] OR "ibuprofen"[All Fields] OR "naproxen"[All Fields] OR "diclofenac"[All Fields] OR "celecoxib"[All Fields] OR "ketorolac"[All Fields] OR "indomethacin"[All Fields] OR "meloxicam"[All Fields] OR "etoricoxib"[All Fields] OR "COX-2 inhibitors"[All Fields]) |
| 9 | ("Dental Implants"[MeSH Terms] OR "dental implant"[All Fields] OR "implant survival"[All Fields] OR "implant failure"[All Fields] OR "peri-implantitis"[All Fields] OR "peri-implant disease"[All Fields] OR "peri-implant mucositis"[All Fields] OR "bone loss"[All Fields] OR "marginal bone loss"[All Fields]) AND ("Proton Pump Inhibitors"[MeSH Terms] OR "PPI"[All Fields] OR "proton pump inhibitor"[All Fields] OR "omeprazole"[All Fields] OR "esomeprazole"[All Fields] OR "pantoprazole"[All Fields] OR "lansoprazole"[All Fields] OR "rabeprazole"[All Fields] OR "dexlansoprazole"[All Fields] OR "Proton Pump Inhibitors"[All Fields]) |
| 10 | ("Dental Implants"[MeSH Terms] OR "dental implant"[All Fields] OR "implant survival"[All Fields] OR "implant failure"[All Fields] OR "peri-implantitis"[All Fields] OR "peri-implant disease"[All Fields] OR "peri-implant mucositis"[All Fields] OR "bone loss"[All Fields] OR "marginal bone loss"[All Fields]) AND ("selective serotonin reuptake inhibitors"[MeSH Terms] OR "SSRI"[All Fields] OR "SSRIs"[All Fields] OR ("selective serotonin reuptake inhibitors"[Pharmacological Action] OR "selective serotonin reuptake inhibitors"[Supplementary Concept] OR "selective serotonin reuptake inhibitors"[All Fields] OR "selective serotonin reuptake inhibitors"[MeSH Terms] OR ("selective"[All Fields] AND "serotonin"[All Fields] AND "reuptake"[All Fields] AND "inhibitors"[All Fields])) OR "fluoxetine"[All Fields] OR "sertraline"[All Fields] OR "paroxetine"[All Fields] OR "citalopram"[All Fields] OR "escitalopram"[All Fields] OR "fluvoxamine"[All Fields]) |

| ***Supplementary table 2.*** *Risk Of Bias In Non-randomized Studies – of Interventions, Version 2 (ROBINS-I*  *V2)* | D1 | D2 | D3 | D4 | D5 | D6 | D7 | Overall |
| --- | --- | --- | --- | --- | --- | --- | --- | --- |
| Anitua et al. (2018) |  |  |  |  |  |  |  |  |
| Altay et al. (2018) |  |  |  |  |  |  |  |  |
| Altay et al. (2019) |  |  |  |  |  |  |  |  |
| Basson et al (2023) |  |  |  |  |  |  |  |  |
| Block et al. (2025) |  |  |  |  |  |  |  |  |
| Brizuela-Velasco et al. (2021) |  |  |  |  |  |  |  |  |
| Carr et al. (2019) |  |  |  |  |  |  |  |  |
| Chandra et al. (2021) |  |  |  |  |  |  |  |  |
| Chaushu et al. (2023) |  |  |  |  |  |  |  |  |
| Chatzopoulos et al. (2025) |  |  |  |  |  |  |  |  |
| Chatzopoulos et al. (2025) |  |  |  |  |  |  |  |  |
| Chrcanovic et al. (2017) |  |  |  |  |  |  |  |  |
| Chrcanovic et al. (2017) |  |  |  |  |  |  |  |  |
| Chrcanovic et al. (2016) |  |  |  |  |  |  |  |  |
| Chrcanovic et al. (2017) |  |  |  |  |  |  |  |  |
| Corbella et al. (2022) |  |  |  |  |  |  |  |  |
| Deepa et al. (2018) |  |  |  |  |  |  |  |  |
| D'Orto et al. (2024) |  |  |  |  |  |  |  |  |
| El-Sherbin et al. (2018) |  |  |  |  |  |  |  |  |
| Galletti at al. (2020) |  |  |  |  |  |  |  |  |
| Gandhi et al. (2019) |  |  |  |  |  |  |  |  |
| Gu et al. (2011) |  |  |  |  |  |  |  |  |
| Hakam et al. (2020) |  |  |  |  |  |  |  |  |
| Hernandez et al. (2019) |  |  |  |  |  |  |  |  |
| Jonas et al. (2024) |  |  |  |  |  |  |  |  |
| Khamis et al. (2019) |  |  |  |  |  |  |  |  |
| Kotsailidi et al. (2022) |  |  |  |  |  |  |  |  |
| Masri et al. (2023) |  |  |  |  |  |  |  |  |
| Masri et al. (2025) |  |  |  |  |  |  |  |  |
| Montebugnoli et al. (2015 |  |  |  |  |  |  |  |  |
| Paredes et al. (2017) |  |  |  |  |  |  |  |  |
| Petsinis et al. (2017) |  |  |  |  |  |  |  |  |
| Radzewski et al. (2019) |  |  |  |  |  |  |  |  |
| Rodríguez-Pena et al. (2022) |  |  |  |  |  |  |  |  |
| Rogoszinski et al. (2023) |  |  |  |  |  |  |  |  |
| Romandini et al. (2021) |  |  |  |  |  |  |  |  |
| Seki et al. (2020) |  |  |  |  |  |  |  |  |
| Tattan et al (2021) |  |  |  |  |  |  |  |  |
| Tonini et al. (2022) |  |  |  |  |  |  |  |  |
| Urdaneta et al. (2011) |  |  |  |  |  |  |  |  |
| Ursomanno et al. (2020) |  |  |  |  |  |  |  |  |
| Winnett et al. (2014) |  |  |  |  |  |  |  |  |
| Wu et al. (2014) |  |  |  |  |  |  |  |  |
| Wu et al. (2017 |  |  |  |  |  |  |  |  |
| Wu et al. (2016) |  |  |  |  |  |  |  |  |

D1: Bias due to confounding

Low

D2: Bias due to selection of participants

Moderate

D3: Bias in classification of interventions

Serious

D4: Bias due to deviations from intented interventions

Critical

D5: Bias due to missing data

D6: Bias in measuement of outcomes

D7: Bias in sleection of the reported result

**Supplementary table 3.** Risk-of-bias tool for randomized trials (RoB 2)

|  | D1 | D2 | D3 | D4 | D5 | Overall |
| --- | --- | --- | --- | --- | --- | --- |
| Alissa et al. (2009) |  |  |  |  |  |  |
| Jeffcoat et al. (1995) |  |  |  |  |  |  |
| Kumchai et al. (2025) |  |  |  |  |  |  |
| Markovic et al. (2017) |  |  |  |  |  |  |
| Reddy et al. (1990) |  |  |  |  |  |  |
| Sakka et al. (2013) |  |  |  |  |  |  |

Low

D1: Bias arising from the randomization process

Some concerns

D2: Bias due to deviations from intended intervention.

D3: Bias due to missing outcome data.

High

D4: Bias in measurement of the outcome.

D5: Bias in selection of the reported result.
